# Digital and artificial intelligence-assisted medication management for older adults with chronic diseases: a systematic review and meta-analysis of randomized controlled trials

**DOI:** 10.3389/fpubh.2026.1956051

**Published:** 2026-10-22

**Authors:** Yuyan Cheng, Weidong Huang, Jianan Tian

**Affiliations:** School of Nursing, Changchun University of Chinese Medicine, Jilin, China

**Keywords:** artificial intelligence, chronic disease, medication management, meta-analysis, older adults

## Abstract

**Objective:**

This systematic review and meta-analysis aims to synthesize the results of randomized controlled trials to determine whether medication management approaches based on digital technologies and artificial intelligence/machine learning can indeed bring measurable clinical improvements in older adults with at least one chronic diseases.

**Methods:**

To identify relevant RCTs, we performed a systematic search of PubMed, Embase, Cochrane Library, CNKI, Wanfang, and other databases from their inception through June 30, 2026, for studies of digital and AI/ML-based medication management in older adults with at least one chronic diseases. All stages of study selection, data extraction, and risk-of-bias assessment were conducted independently by two reviewers, applying the Cochrane RoB 2.0 tool. Meta-analytic pooling was conducted via RevMan 5.4.

**Results:**

Nineteen RCTs met the inclusion criteria, of which 6 were included in the meta-analysis and 13 in the descriptive synthesis. For medication adherence, three studies reported highly heterogeneous results (*I*^2^ = 97%), and because of this heterogeneity and the small number of studies, the data were not pooled; the individual study estimates ranged from SMD = −0.28–SMD = 1.89. For PIMs, only one study reported a comparable outcome; no pooled estimate was calculated. For hospitalization and mortality, the two included studies used different effect measures (HR and OR) and were therefore presented separately: Blum et al. reported an HR of 0.95 (95% CI 0.77–1.17), and Rieckert et al. reported an OR of 0.88 (95% CI 0.73–1.07).

**Conclusion:**

The current body of evidence does not establish clinically meaningful benefits of digital or AI/ML-based medication management on hard endpoints such as hospitalization and mortality among older adults with at least one chronic diseases. For medication adherence, the evidence remains inconclusive and highly heterogeneous, and no reliable pooled estimate could be obtained. Given the pronounced heterogeneity and the limited pool of high-quality RCTs, current evidence is insufficient to establish clinical benefit for the routine implementation of digital or AI/ML-based medication management systems in geriatric care and further rigorously designed studies are warranted.

**Systematic review registration:**

CRD420261460445.

## 1 Introduction

The global population is undergoing accelerated aging, with the proportion of individuals aged ≥60 years projected to climb from 12% in 2015 to 22% by 2050. Surveys indicate that the majority of older adults are affected by chronic conditions, with a considerable proportion having more than one comorbidity (1). Among older patients with chronic illnesses, estimates suggest that 40–60% of those aged ≥65 years are on five or more concurrent medications concurrently (2). Polypharmacy may predispose patients to drug–drug interactions and adverse events, while substantially reducing treatment adherence, thereby leading to unfavorable patient-relevant outcomes, such as heightened readmission rates, diminished quality of life, and escalating healthcare costs (3). In this context, artificial intelligence (AI) is increasingly recognized as a valuable tool for enabling more accurate and personalized pharmacotherapy management (4). In this review, digital medication management is defined as interventions delivered through digital platforms such as smartphone applications, SMS, wearable sensors, or automated pharmacy systems. AI/ML-based medication management is defined as a subset of digital interventions that additionally use machine learning, deep learning, natural language processing, knowledge graphs, or adaptive algorithms to support medication review, risk prediction, personalized recommendation, or interactive counseling. These systems differ from rule-based clinical decision support systems (CDSS), which only execute pre-specified guideline rules, and from conventional mHealth interventions such as simple short-message reminders or non-interactive educational videos. AI/ML-based medication management primarily functions through machine learning models that predict individualized medication risks, AI-powered chatbots that provide medication counseling and health education, and adaptive algorithms that personalize medication review. Rule-based CDSS that only execute pre-specified guideline rules were classified separately and were not considered AI/ML-based interventions (5). Compared with traditional medication management approaches, AI systems offer advantages such as real-time responsiveness, personalized interventions, and scalability scalable deployment, making them particularly suitable for older adults with chronic conditions. The medication management scenarios of older adults with chronic conditions. Previous reviews have examined AI in medication management for older adults (6) and compared AI with rule-based decision support (7). In addition, a recent economic evaluation of AI in medication management should be considered (8). However, these reviews either included non-RCT designs or did not quantitatively pool RCT evidence. The present review focuses exclusively on RCTs, distinguishes AI/ML-based interventions from rule-based CDSS and conventional mHealth, and provides a quantitative synthesis of clinical outcomes. However, the clinical urgency for such interventions is underscored by recent real-world evidence. Large-scale studies have documented a substantial burden of potentially inappropriate medication (PIM) prescribing in outpatient geriatric populations (9), while real-time electronic prescribing alerts have been associated with reductions in PIM prescribing (10, 11). These findings highlight the pressing need for effective medication-management strategies and provide a real-world context for interpreting the RCT evidence synthesized in this review.

## 2 Methods

### 2.1 Protocol and registration

This systematic review was prospectively registered with PROSPERO (CRD420261460445) on 24 July 2026. The protocol was developed in accordance with the PRISMA-P guidelines, and the review was conducted and reported according to the PRISMA 2020 statement. Because the registration date was after the final search date, the review should be regarded as retrospectively registered.

### 2.2 Literature search

We performed a comprehensive literature search across multiple electronic databases, covering both English and Chinese sources. The English-language databases queried included PubMed, Embase, Cochrane library, web of science, and IEEE Xplore; the Chinese repositories comprised CNKI, Wanfang, VIP, and CBM. The search period extended from database inception through June 30, 2026. A combination of MeSH terms and free-text keywords was used to optimize retrieval sensitivity. The full search strategies for each database are provided in Supplementary Table S1. In addition, we searched ClinicalTrials.gov, WHO ICTRP, and ProQuest Dissertations and Theses to identify ongoing trials and grey literature. A total of 25 records were retrieved from these sources. After screening, no additional eligible RCTs were identified because the retrieved records were ongoing or unpublished trials without available outcome data, or did not meet the predefined eligibility criteria. The detailed search strategy employed in PubMed is shown in Figure 1.

**Figure 1:**
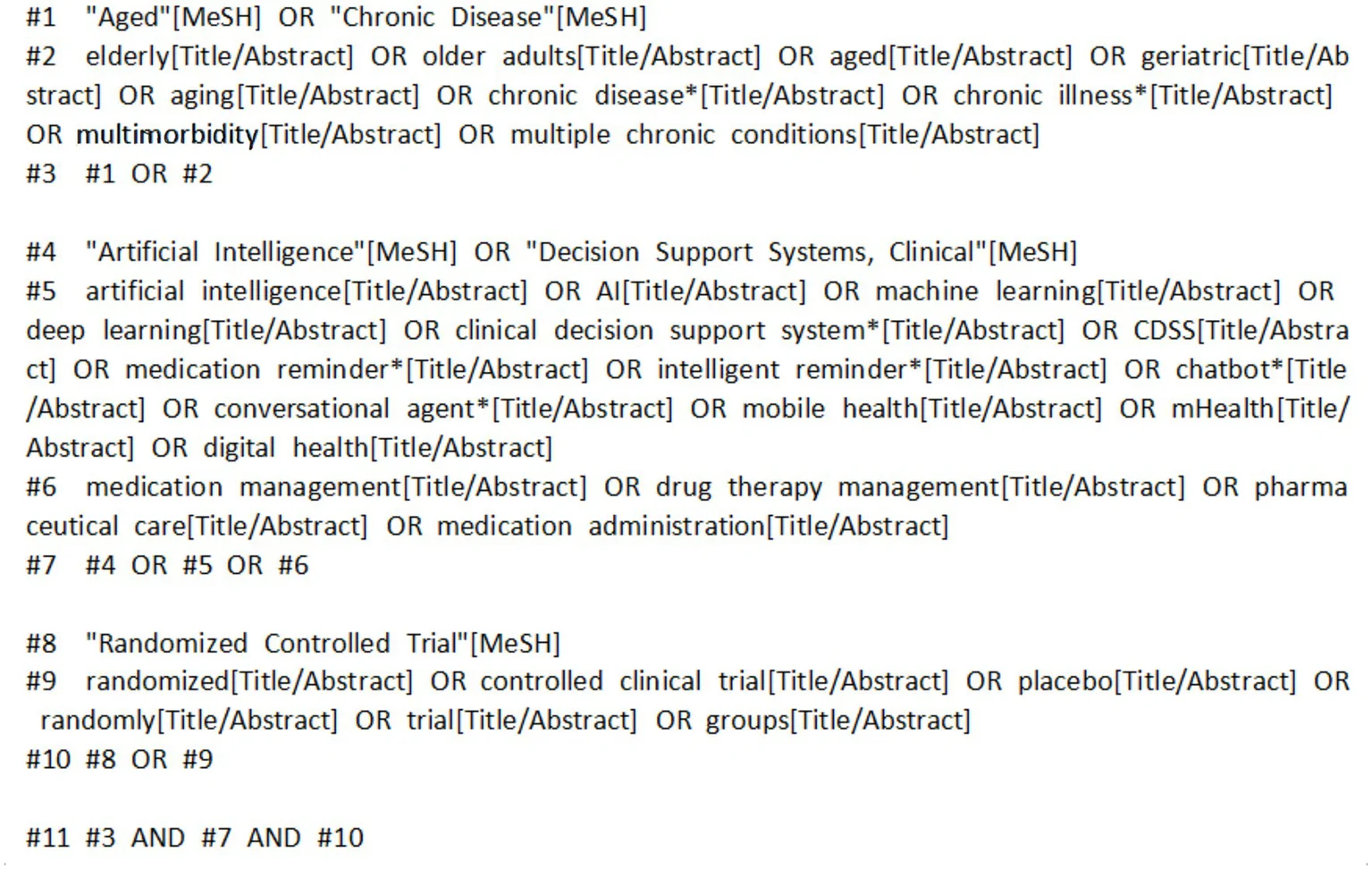
Literature search strategy (PubMed).

### 2.3 Study selection criteria

#### 2.3.1 Inclusion criteria

Studies qualified for inclusion when they met the following criteria:

##### 2.3.1.1 Study design

Randomized controlled trials (RCTs) that assessed the role of digital or AI/ML-based medication management in older adults with chronic conditions.

##### 2.3.1.2 Participants

Participants aged ≥60 years who had at least one confirmed chronic diseases, defined according to validated clinical guidelines (e.g., the Chinese Guidelines for Prevention and Treatment of Hypertension (12) and the Chinese Guidelines for Type 2 Diabetes (13)), and who required long-term pharmacological therapy (≥1 regular medication). One study with an age threshold of ≥55 years was included because the mean age was ≥60 years.

##### 2.3.1.3 Intervention

The experimental arm incorporated a digital or AI/ML-based medication management intervention, Digital interventions were defined as those delivered through digital platforms such as smartphone applications, SMS, wearable sensors, or automated pharmacy systems. AI/ML-based interventions were defined as digital systems that additionally use at least one of the following techniques: machine learning, deep learning, natural language processing, knowledge graph, or adaptive/self-learning algorithms. Rule-based CDSS that only execute pre-specified guideline rules, simple short-message reminders, and non-interactive educational videos were classified as conventional mHealth and analyzed separately.

##### 2.3.1.4 Outcomes

Included at least one of the following: medication adherence, incidence of PIMs, adverse drug events, disease-specific clinical physiological indicators, hospitalization rates, or quality of life.

#### 2.3.2 Exclusion criteria

We excluded records that satisfied any of the following criteria: (a) duplicate publications; (b) unavailable full texts with no response from authors after attempted contact; (c) published in languages other than English or Chinese; or (d) categorized as conference abstracts, narrative reviews, case reports, study protocols, or other non-empirical research contributions.

#### 2.3.3 Classification of interventions

Included interventions were classified into three categories: (1) AI/ML-based interventions (e.g., machine learning risk prediction, intelligent chatbot, adaptive algorithm); (2) Rule-based CDSS (e.g., STRIPA, OPERAM, OPTICA); (3) Conventional mHealth interventions (e.g., SMS reminders, WeChat video, simple app reminders). The primary analysis included all digital and AI/ML-based interventions. Studies were categorized by intervention type for descriptive purposes.

### 2.4 Study selection and data extraction process

Two independent reviewers screened all retrieved records in a two-step process: initially, they assessed titles and abstracts; subsequently, they evaluated the full texts of potentially eligible studies. We used EndNote to identify and remove duplicate records before screening, and manually verified the deduplication results. The two reviewers discussed any discrepancies until they reached agreement, with unresolved disagreements referred to a third reviewer.

For each eligible study, we extracted a standardized set of information:

*General study characteristics*: first author, publication year, country of origin, and study design;

*Participant demographics*: sample size, age range, sex distribution, chronic disease type, and baseline medication count;

*Intervention specifications*: AI tool category, delivery protocol, frequency, total duration, and control group procedures;

*Outcome-related data*: specific outcome name, measurement instrument or scale, follow-up assessment points, and quantitative information (sample sizes, means, standard deviations, or event frequencies) required for effect size pooling.

### 2.5 Risk of bias evaluation

We appraised the methodological quality of each included trial using the Cochrane Risk of Bias 2.0 (RoB 2.0) instrument. This tool evaluates five key domains: the randomization procedure; departures from the intended intervention; completeness of outcome data; outcome measurement validity; and selective outcome reporting. Two reviewers independently screened the risk-of-bias assessment and verified their judgments through cross-checking. The two reviewers first attempted to resolve any discrepancies through discussion. If agreement could not be reached, a third reviewer was consulted. Each study was rated as “low risk of bias,” “some concerns,” or “high risk of bias” according to the RoB 2.0 framework. Studies rated as high risk of bias were excluded from the quantitative synthesis and considered only in the descriptive summary.

### 2.6 Statistical synthesis

All meta-analytic computations were carried out using RevMan 5.4. For continuous outcomes, we estimated the pooled effect as the mean difference (MD) or standardized mean difference (SMD), depending on whether the same instrument was used across studies. For dichotomous outcomes, we employed the odds ratio (OR) or risk ratio (RR). When studies reported different effect measures (e.g., HR, OR, RR), we either converted them to a common effect measure using the Cochrane-recommended formulas, or, when reliable conversion was not possible, we presented the results separately without pooling. We used the Chi-square test and I^2^ statistic to assess between-study heterogeneity. For the medication adherence outcome, due to substantial heterogeneity (*I*^2^ = 97%) and the small number of studies (*n* = 3), we did not pool the data and instead presented the results descriptively. Subgroup analyses were performed by intervention type, disease type, and baseline adherence level. Due to the limited number of studies and substantial heterogeneity, sensitivity analyses were not performed.

### 2.7 Certainty of evidence assessment

The certainty of evidence for each outcome was assessed using the Grading of Recommendations Assessment, Development and Evaluation (GRADE) approach. Two reviewers independently evaluated the risk of bias, inconsistency, indirectness, imprecision, and publication bias for each outcome. The certainty of evidence was categorized as high, moderate, low, or very low. Discrepancies were resolved through discussion with a third reviewer.

## 3 Results

### 3.1 Study selection

A total of 1,002 records were identified through database searching. After sequential screening, 80 reports were assessed for eligibility. Of these, 61 reports were excluded, including 29 studies rated as high risk of bias during the full-text screening. Finally, 19 RCTs were included in the systematic review (14–32). Of these, 6 studies provided complete effect size data for meta-analysis, while the remaining 13 were included in descriptive synthesis only due to data unavailability for standardized effect size extraction. The study selection process is illustrated in the PRISMA flow diagram (Figure 2).

**Figure 2:**
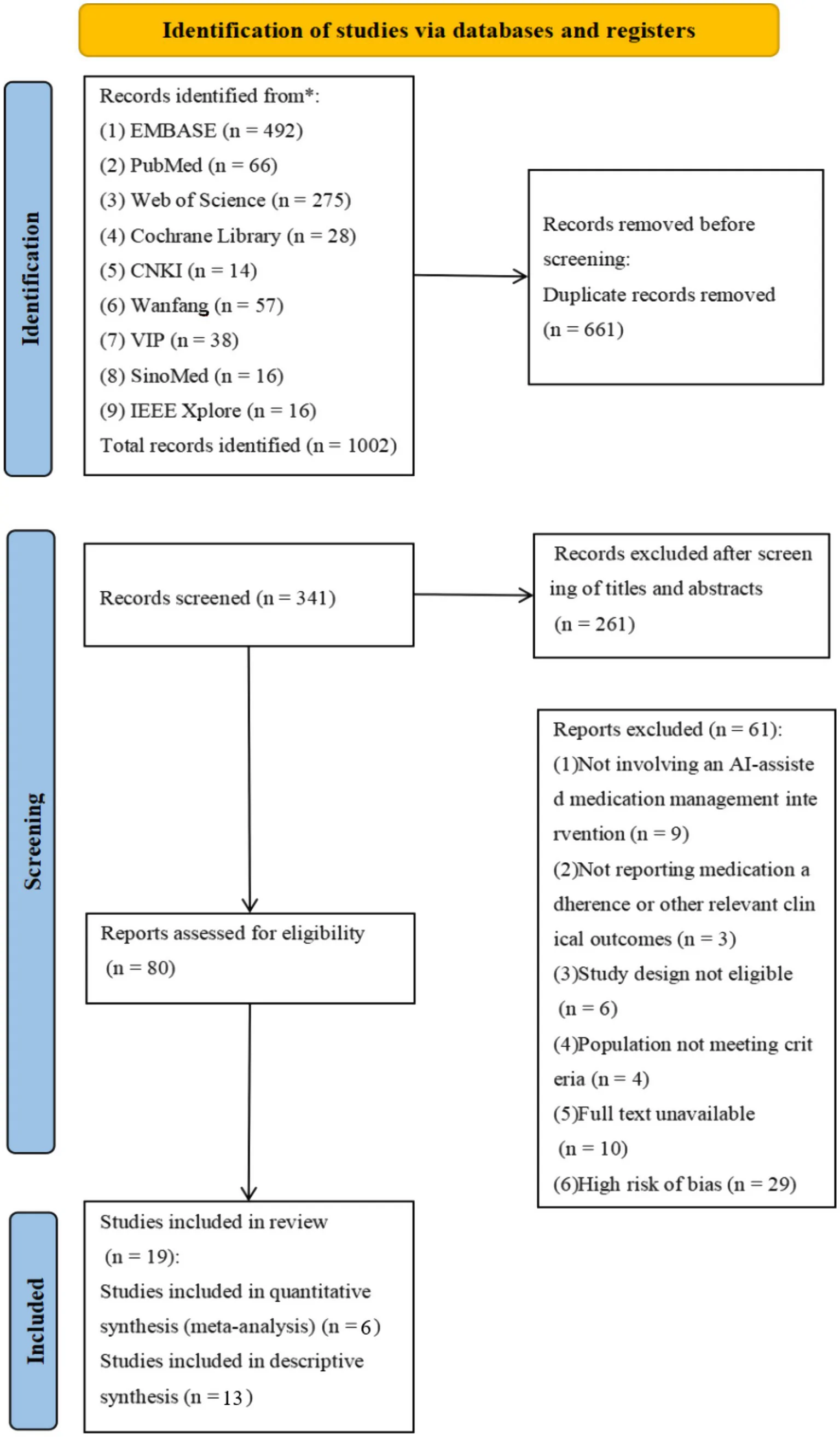
PRISMA flow diagram.

### 3.2 Characteristics of included studies

The 19 RCTs that met our inclusion criteria spanned a 21-year publication window (2005–2026) and collectively involved 6,876 participants (3,466 allocated to the intervention arms vs. 3,410 to the control groups). These trials were distributed across nine countries, with the majority (*n* = 8) carried out in the United States.2 each from India and China, and 1 each from Switzerland, Germany, Turkey, Iran, Finland, and a multi-country collaboration. Participants were older adults with chronic diseases, with age thresholds ranging from ≥55 to ≥70 years. Disease types included type 2 diabetes mellitus (4 studies), hypertension (3 studies), chronic heart failure (2 studies), atrial fibrillation, hyperlipidemia, asthma/COPD, and renal insufficiency (1 each), and multiple chronic conditions (6 studies). Given the substantial clinical heterogeneity across disease categories, subgroup analyses by disease type were performed for the medication adherence outcome. However, because most outcome categories contained fewer than three studies per disease type, these subgroup analyses were exploratory and should be interpreted with caution. According to the predefined classification, 2 studies were classified as AI/ML-based interventions, 8 as rule-based CDSS, and 9 as conventional mHealth interventions. Intervention durations ranged from 3 to 12 months: 3 studies for 3 months, 11 studies for 6 months, and 5 studies for 12 months (Table 1).

**Table 1:** Baseline characteristics of included studies.

Study	Year	Country	Study design	Sample size (I/C)	Age (years)	Chronic disease type	Intervention	Intervention category	Control	Intervention duration	Outcomes
Smith et al. (14)	2008	USA	RCT	128/122	≥65	Diabetes mellitus	CDSS	Rule-based CDSS	Usual care	12 months	①③
Tierney et al. (15)	2005	USA	RCT	215/210	≥60	Asthma/COPD	CDSS	Rule-based CDSS	Usual care	12 months	①
Subramanian et al. (16)	2012	USA	RCT	89/85	≥65	Renal insufficiency	CDSS	Rule-based CDSS	Usual care	6 months	②④
Nietert et al. (17)	2009	USA	RCT	312/308	≥60	Chronic disease (multiple)	Automated pharmacy intervention	Rule-based CDSS	Usual care	12 months	③⑤
Fried et al. (18)	2017	USA	RCT	98/102	≥65	Polypharmacy	CDSS(TRIM)	Rule-based CDSS	Usual care	6 months	②
Kleinmanet al (19)	2017	India	RCT	150/150	≥60	Type 2 diabetes	mHealth APP	Conventional mHealth	Usual care	6 months	①③
Moorheadet al (20)	2017	USA	RCT	200/196	≥60	Chronic disease (multiple)	Smart reminder + sensor	Conventional mHealth	Usual care	3 months	①
Dugas et al. (21)	2018	USA	RCT	65/67	≥55*	Type 2 diabetes	mHealth APP	Conventional mHealth	Usual care	6 months	①③
Blum et al. (22)	2021	Multi-country	RCT	963/1045	≥70	Chronic disease (multiple)	CDSS(STRIPA/OPERAM)	Rule-based CDSS	Usual care	12 months	⑤
Rieckert et al. (23)	2020	Germany	RCT	251/249	≥65	Chronic disease (multiple)	CDSS	Rule-based CDSS	Usual care	6 months	①③
Jungo et al. (24)	2023	Switzerland	RCT	206/204	≥65	Chronic disease (multiple)	CDSS(OPTICA)	Rule-based CDSS	Usual care	12 months	②⑤
Buis et al. (25)	2024	USA	RCT	183/177	≥60	Hypertension	mHealth APP(MI-BP)	Conventional mHealth	Usual care	6 months	①③
Xu et al. (26)	2024	China	RCT	235/228	≥60	Atrial fibrillation	Smartphone APP	Conventional mHealth	Usual care	6 months	①
Arabaci et al. (27)	2025	Turkey	RCT	45/45	≥65	Hypertension	Smartphone APP	Conventional mHealth	Usual care	3 months	①③
Kitsiou et al. (28)	2025	USA	RCT	110/108	≥60	Chronic heart failure	mHealth APP	Conventional mHealth	Usual care	6 months	①③⑤
Worrall et al. (29)	2025	USA	RCT	156/152	≥60	Chronic disease (multiple)	AI-supported system	AI/ML-based	Usual care	6 months	①②
Mahdian et al. (30)	2026	India	RCT	85/83	≥60	Hyperlipidemia	SMS intervention	Conventional mHealth	Usual care	3 months	①③
Mehta et al. (31)	2026	India	RCT	120/118	≥60	Chronic disease (multiple)	Mobile APP	AI/ML-based	Usual care	6 months	①③⑤
Wang et al. (32)	2026	China	RCT	168/162	≥65	Type 2 diabetes	Mobile video APP	Conventional mHealth	Usual care	6 months	①③

AI/ML, Artificial Intelligence/Machine Learning; CDSS, Clinical Decision Support System; mHealth, Mobile Health. “AI/ML-based” refers to interventions using machine learning, deep learning, or NLP; “Rule-based CDSS” refers to systems executing pre-specified guideline rules; “Conventional mHealth” refers to standard digital tools like SMS, simple apps, or educational videos without AI algorithms. I, intervention group; C, control group. Outcomes: ① medication adherence; ② potentially inappropriate medications (PIMs); ③ blood pressure/blood glucose and other clinical indicators; ④ adverse drug events; ⑤ quality of life/hospitalization rate. *Dugas et al. used an age threshold of ≥55 years; because the mean age was ≥60 years, the study was included but was not included in the meta-analysis because the data were not pooled.

### 3.3 Quality assessment

Using the Cochrane RoB 2.0 tool, among the 19 included studies, 4 studies (16, 17, 26, 30) were rated as low risk of bias, 15 (14, 15, 18–25, 27–29, 31, 32) as some concern., No studies were rated as high risk of bias. The primary source of bias was the domain “deviations from intended interventions,” which is unavoidable in behavioral interventions such as apps and CDSS. Given the large number of studies with some concerns, the overall quality of the included evidence should be interpreted with caution (Table 2).

**Table 2:** Quality assessment of included studies using Cochrane RoB 2.0.

Study	Randomization process (D1)	Deviations from intended interventions (D2)	Missing outcome data (D3)	Measurement of outcome (D4)	Selective reporting (D5)	Quality grade
Arabaci et al. (27)	+	?	+	?	+	Some concerns
Blum et al. (22)	+	?	+	+	+	Some concerns
Buis et al. (25)	+	?	?	+	+	Some concerns
Dugas et al. (21)	+	?	?	+	+	Some concerns
Fried et al. (18)	?	?	?	?	+	Some concerns
Jungo et al. (24)	+	?	+	+	+	Some concerns
Kitsiou et al. (28)	+	?	?	+	+	Some concerns
Kleinman et al. (19)	+	?	+	+	+	Some concerns
Mahdian et al. (30)	+	+	+	+	+	Low risk
Mehta et al. (31)	+	?	+	+	+	Some concerns
Moorhead et al. (20)	?	?	+	?	+	Some concerns
Nietert et al. (17)	+	+	+	+	+	Low risk
Rieckert et al. (23)	+	?	+	+	+	Some concerns
Smith et al. (14)	+	?	+	+	+	Some concerns
Subramanian et al. (16)	+	+	+	+	+	Low risk
Tierney et al. (15)	+	?	+	+	+	Some concerns
Wang et al. (32)	+	?	+	+	+	Some concerns
Worrall et al. (29)	?	?	?	?	+	Some concerns
Xu et al. (26)	+	+	+	+	+	Low risk

+, low risk of bias;?, some concerns; −, high risk of bias.

### 3.4 Meta-analysis results

#### 3.4.1 Medication adherence

Three RCTs evaluated the effect of digital and AI/ML-based medication management on adherence in older adults with chronic diseases (26, 31, 32). Due to substantial heterogeneity (*I*^2^ = 97%) and the small number of studies, we did not pool the data. Xu et al. reported SMD = 1.89 (95% CI 1.41–2.38), Mehta et al. reported SMD = −0.28 (95% CI −0.49 to −0.07), and Wang et al. reported SMD = 0.41 (95% CI 0.07–0.75). The direction and magnitude varied substantially across studies, reflecting differences in intervention type, target disease, and baseline adherence levels. The adherence results should therefore be interpreted with caution and do not provide reliable evidence of a beneficial effect (Figure 3).

**Figure 3:**
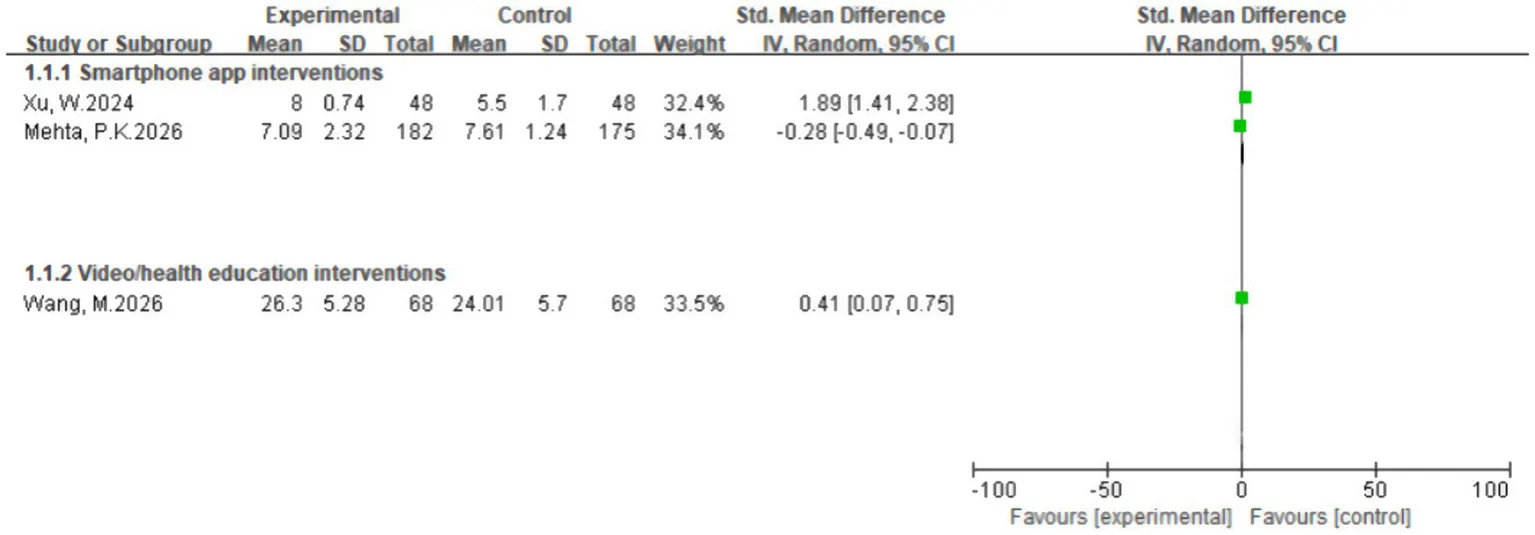
Individual study and subgroup estimates of digital and AI/ML-based medication management on medication adherence in older adults with chronic diseases. Because of substantial heterogeneity (*I*^2^ = 97%), the data were not pooled. The figure displays individual study estimates and subgroup estimates only.

#### 3.4.2 Potentially inappropriate medications

For PIMs, two studies reported relevant outcomes (24, 29). Jungo et al. (24) and Worrall et al. (29). However, because Worrall et al. (29) reported data that were not comparable for pooling, only the OPTICA trial (24) provided a comparable outcome. Jungo et al. (24) reported an OR for MAI score improvement at 12 months (OR = 1.05, 95% CI 0.59–1.87). Consequently, because only one study reported a comparable PIMs outcome, no pooled estimate was calculated (Figure 4).

**Figure 4:**
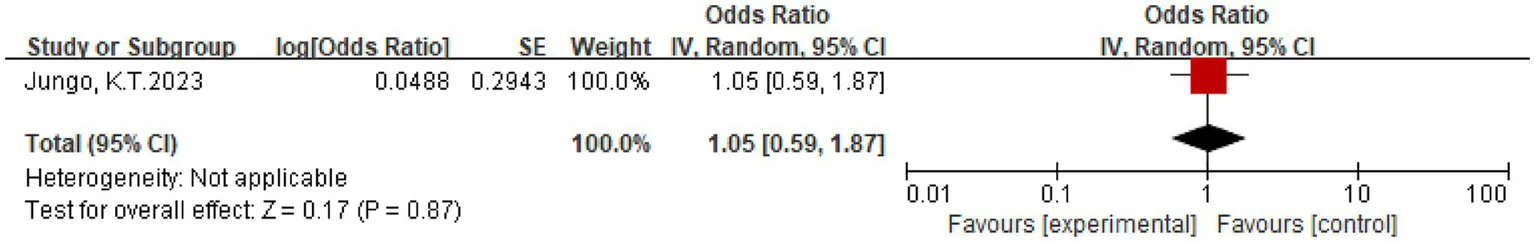
Individual study estimate for PIMs in the OPTICA trial. Jungo et al. reported an OR of 1.05 (95% CI 0.59–1.87) for medication appropriateness. Because only one study reported a comparable outcome, no pooled estimate was calculated. OR, odds ratio; CI, confidence interval.

#### 3.4.3 Hospitalization and mortality

Two studies assessed the effect of CDSS-assisted medication review on hospitalization or mortality in older adults with chronic diseases (22, 23). Because the two studies used different effect measures (HR and OR), they were not pooled and are presented separately. Blum et al. (22) (OPERAM trial) reported an HR for drug-related hospitalization at 12 months (HR = 0.95, 95% CI 0.77–1.17); Rieckert et al. (23) (PRIMA-eDS trial) reported an OR for unplanned hospitalization or mortality at 24 months (OR = 0.88, 95% CI 0.73–1.07) (Figure 5).

**Figure 5:**
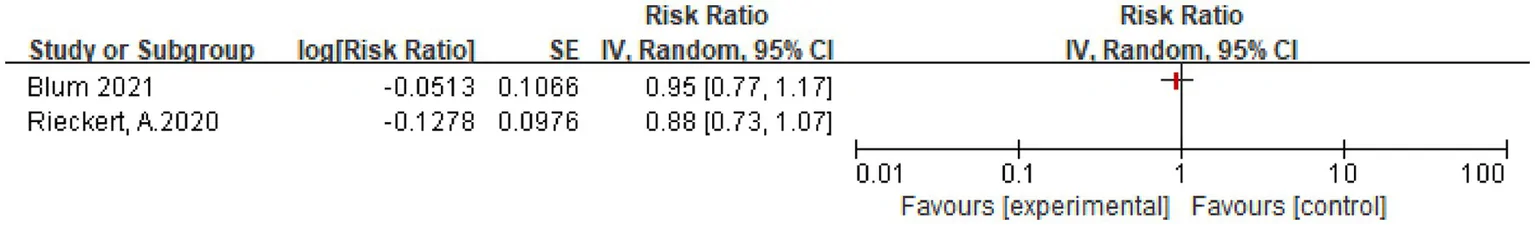
Individual study estimates for hospitalization and mortality in older adults with chronic diseases. Blum et al. reported a hazard ratio (HR) of 0.95 (95% CI 0.77–1.17) for drug-related hospitalization at 12 months. Rieckert et al. reported an odds ratio (OR) of 0.88 (95% CI 0.73–1.07) for unplanned hospitalization or mortality at 24 months. The original RevMan output displayed a generic “Risk Ratio” label and an “IV, Random” model; because the two studies used different effect measures, no pooled estimate was calculated. HR, hazard ratio; OR, odds ratio; CI, confidence interval.

#### 3.4.4 Sensitivity analysis

Formal sensitivity analyses were not performed for any outcome due to the limited number of contributing studies (*n* = 3 for adherence, *n* = 1 for PIMs, *n* = 2 for hospitalization/mortality) and substantial heterogeneity. The results should be interpreted with caution given these limitations (Table 3).

**Table 3:** Summary of outcomes and reasons for not pooling.

Outcome	Number of studies	Reason for not pooling	Reported effect estimates
Medication adherence	3	*I*^2^ = 97%; substantial heterogeneity	Xu et al. SMD = 1.89 (1.41–2.38); Mehta et al. SMD = −0.28 (−0.49 to −0.07); Wang et al. SMD = 0.41 (0.07–0.75)
PIMs	1	Only one study reported a comparable outcome	Jungo et al. OR = 1.05 (0.59–1.87)
Hospitalization/mortality	2	Different effect measures (HR and OR)	Blum et al. HR = 0.95 (0.77–1.17); Rieckert et al. OR = 0.88 (0.73–1.07)

SMD, standardized mean difference; OR, odds ratio; CI, confidence interval; “—” indicates that only one study remained after exclusion, so I^2^ could not be calculated.

#### 3.4.5 Publication bias assessment

We did not perform formal publication bias tests (e.g., funnel plots or egger’s regression) for any outcome, because each pooled analysis included fewer than 10 studies—the commonly suggested threshold for such diagnostics, making such tests statistically underpowered and potentially misleading. Instead, we assessed the risk of selective reporting by reviewing trial registration information for each included study. Notably, the more recent RCTs incorporated in this review were prospectively registered in publicly accessible platforms such as ClinicalTrials.gov. Although a small number of earlier trials lacked explicit registration details, our comprehensive search across both English and Chinese databases—covering major sources of grey literature and region-specific journals—likely minimized the extent to which publication bias might have affected the summary estimates.

#### 3.4.6 Certainty of evidence

The certainty of evidence was assessed using the GRADE approach and is summarized in Table 4. The overall certainty of evidence was very low for medication adherence, primarily due to serious inconsistency (*I*^2^ = 97%) and imprecision (small number of studies). For potentially inappropriate medications (PIMs) and hospitalization/mortality, the certainty of evidence was low, downgraded mainly for serious imprecision (single study or different effect measures) and risk of bias concerns.

**Table 4:** GRADE certainty of evidence for pooled outcomes in the meta-analysis.

Outcome	Number of studies (participants)	Effect estimate (95% CI)	Certainty of evidence (GRADE)	Reasons for downgrading
Medication adherence	3 RCTs (589)	Not pooled due to heterogeneity	Very low	Serious inconsistency (*I*^2^ = 97%*I*^2^ = 97%), serious imprecision (small number of studies), and risk of bias (some concerns)
Potentially inappropriate medications (PIMs)	1 RCT (410)	OR = 1.05 (0.59–1.87)	Low	Serious imprecision (only one study, wide confidence interval)
Hospitalization/mortality	2 RCTs (2,508)	Blum: H*R* = 0.95 (0.77–1.17) Rieckert: O*R* = 0.88 (0.73–1.07)	Low	Serious imprecision (different effect measures, few events), and risk of bias (some concerns)

GRADE, Grading of Recommendations Assessment; Development and Evaluation; RCT, randomized controlled trial; OR, odds ratio; HR, hazard ratio; CI, confidence interval. Certainty of evidence categories: High, Moderate, Low, Very Low. The “Low” and “Very Low” certainty of evidence reflects the inherent limitations of the existing primary studies (e.g., high heterogeneity, small sample sizes, and risk of bias), rather than a definitive lack of efficacy of digital/AI interventions. These findings highlight a critical evidence gap and call for more rigorous, standardized RCTs in this field.

### 3.5 Descriptive synthesis

According to the predefined classification, 2 studies were classified as AI/ML-based interventions, 8 as rule-based CDSS, and 9 as conventional mHealth interventions. Of the 19 included RCTs, 6 studies were included in the meta-analysis, and the remaining 13 were narratively synthesized, organizing them by thematic focus.

#### 3.5.1 Medication adherence

Five trials provided data on the impact of digital and AI/ML-based medication management on adherence outcomes (18–20, 29, 30). Among these, Kleinman et al. (19) evaluated a conventional mHealth APP, Worrall et al. (29) evaluated an AI pharmacist-supported adherence program, Mahdian et al. (30) evaluated SMS reminders (conventional mHealth), Moorhead et al. (20) evaluated a smart reminder with sensor (conventional mHealth), and Fried et al. (18) evaluated the TRIM tool (rule-based CDSS). At 6 months post-intervention, the adherence improvement rate in Kleinman et al. (19) trial was 12.8% among controls, compared with 39.0% among those receiving the mHealth intervention (*p* = 0.03). Worrall et al. (29) showed that medication possession ratios (MPR) for hypertension, hyperlipidemia, and diabetes patients increased by 5.9%, 7.9%, and 6.4%, respectively. Mahdian et al. (30) found that daily SMS reminders significantly improved Morisky adherence scores from 3.83 to 6.20 after 6 weeks (*p* < 0.001), while the comparative group showed minimal within-group variation. Moorhead et al. (20) reported that patients who saw smart reminders had a medication-taking rate of 75% ± 18%, significantly higher than 59% ± 24% when reminders were not seen (*p* < 0.001). Fried et al. (18) found that the TRIM tool corrected 48.4% of medication discrepancies in the intervention group versus 14.3% in the control group (*p* < 0.001), though no significant effect on PIM count or medication number was observed.

#### 3.5.2 Clinical indicators

Three studies reported on the effect of AI-assisted medication management on clinical indicators (14, 15, 25). Buis et al. (25) (MI-BP trial) evaluated a conventional mHealth APP and found no significant between-group difference in systolic blood pressure reduction (intervention: −22.5 mmHg; control: −24.1 mmHg; *p* = 0.99). Smith et al. (14) evaluated a rule-based CDSS and revealed no significant between-group difference, with an adjusted mean difference of −1% (95% CI, −19 to 17%). Tierney et al. (15) evaluated a rule-based CDSS and found no significant improvement in clinical outcomes for asthma/COPD, with guideline adherence rates of 32% in both groups.

#### 3.5.3 Other outcomes

Four studies reported other outcomes (16, 17, 21, 27). Dugas et al. (21) evaluated a conventional mHealth APP used a points-based adherence score (non-standardized, not included in meta-analysis), but noted that the high-adherence group showed a 1.07% reduction in HbA1c (*p* < 0.01). Nietert et al. (17) evaluated an automated pharmacy intervention and found no significant improvement in chronic disease prescription refill persistence. Subramanian et al. (16) evaluated a rule-based CDSS and reported limited immediate direct cost savings. Arabaci et al. (27) evaluated a conventional mHealth APP and reported that app-supported nursing care improved medication adherence in hypertensive patients, though effect sizes were not reported.

Collectively, these descriptive findings indicate that digital medication management has a positive trend in improving medication adherence, with all 5 studies reporting positive effects across various intervention types (apps, SMS, sensors, AI pharmacist support). However, the 3 studies by Buis et al. (25), Smith et al. (14) and Tierney et al. (15) showed no significant clinical benefit such as blood pressure or metabolic parameters, suggesting that adherence improvement may not directly translate into clinical benefit. Our results align with those reported by Chen et al. (33) on AI chatbots, which also noted high heterogeneity and a weak evidence base in the AI medication management field, highlighting the need for more high-quality, homogeneous studies. Chen et al. (33) was published after the search date and was used only for background and contextual discussion, not for eligibility assessment.

## 4 Discussion

Drawing on data from 19 RCTs, our analysis offers a comparative synthesis of digital and AI/ML-based medication management across diverse technological platforms in the geriatric chronic disease population. Because the majority of included interventions were conventional mHealth or rule-based CDSS rather than genuine AI/ML, the findings should be attributed to the broader category of digital medication management rather than to AI alone. The core findings indicate a positive trend in medication adherence with digital interventions but with uncertain evidence, while no significant benefits were observed for PIMs, hospitalization, or mortality. The PIM and hospitalization/mortality analyses included only one and two studies, respectively, and neither demonstrated that digital or AI/ML-based interventions significantly improved these patient-important outcomes.

For medication adherence, the three included studies reported highly heterogeneous results (*I*^2^ = 97%), and we therefore did not pool the data. The individual study estimates ranged from SMD = −0.28 Mehta et al. (31) to 1.89 Xu et al. (26). The heterogeneity stemmed more from differences in target diseases and baseline adherence levels, and intervention type. The APP subgroup included only 2 studies and the video subgroup only 1, resulting in insufficient statistical power. The meta-analysis by Chen et al. (33) on AI chatbots similarly reported *I*^2^ = 97%, suggesting that high heterogeneity is a common phenomenon in this field. The substantial heterogeneity (*I*^2^ = 97%) likely reflects inherent differences in interventions, target diseases, and baseline characteristics across studies. Therefore, aggregating “digital and AI/ML-based medication management” as a single category for meta-analysis may be inappropriate.

For PIMs, only one study reported a comparable outcome, so no pooled estimate was calculated. For hospitalization and mortality, the two included studies used different effect measures and were therefore presented separately. Given the extremely limited number of studies (*n* = 1 for PIMs and *n* = 2 for hospitalization/mortality), the evidence for these outcomes is insufficient to draw definitive conclusions. Neither outcome reached statistical significance. Clinical inertia, mistrust of digital or AI/ML-based recommendations, and the limited effect of a single intervention are possible explanations for the limited implementation, but these mechanisms were not directly measured in the included RCTs and should be regarded as hypotheses rather than established causes.

In summary, current evidence does not support that digital or AI/ML-based medication management significantly improves hospitalization and mortality in older adults with chronic diseases. Although digital or AI/ML-based interventions shows initial promise in improving medication adherence and has certain advantages in identifying PIMs, these findings have not translated into definitive benefits in hard clinical endpoints, and the evidence remains characterized by substantial heterogeneity. There is a complex translational gap between “identifying problems” and “improving outcomes.” Given the high heterogeneity and weak evidence base in this field, clinicians should interpret claims of “effectiveness” of digital and AI/ML-based medication management tools with caution. The current evidence is insufficient to establish clinical benefit for integrating digital or AI/ML-based medication management into routine care in geriatric cohorts affected by chronic conditions. Priorities for upcoming work include large-sample, long-follow-up RCTs in more homogeneous populations, with attention to strategies for increasing uptake of digital or AI/ML-based recommendations and integration into clinical workflows to bridge the translational gap between identification and action. From the evidence assembled in the present review, several clinically relevant observations emerge: 1. Current evidence does not support widespread implementation of digital or AI/ML-based medication management tools for improving hard clinical outcomes in older adults with chronic diseases. Clinicians should interpret claims of effectiveness with caution. 2. Digital or AI/ML-based tools may be considered as adjunctive aids for identifying potentially inappropriate medications (PIMs), but their impact on hospitalization and mortality remains unproven in this population. 3. Future implementation should focus on strategies to increase clinician and patient uptake of digital or AI/ML-based recommendations, as the gap between “identification of medication problems” and “improvement of clinical outcomes” represents a critical translational bottleneck.

Limitations of the evidence base. The overall certainty of evidence in this review was graded as low to very low. This is primarily attributable to substantial heterogeneity across studies (e.g., diverse intervention types and baseline characteristics), imprecision arising from small sample sizes and the limited number of RCTs contributing to each pooled outcome, and methodological concerns regarding the risk of bias. Consequently, the current findings should be interpreted with caution. These limitations highlight a critical evidence gap and underscore the urgent need for future adequately powered, methodologically rigorous RCTs with standardized intervention protocols and consistent outcome measures.

## 5 Conclusion

The evidence concerning digital and AI/ML-based medication management strategies across diverse technological platforms in geriatric populations with chronic diseases was synthesized in the current analysis. Nineteen RCTs were included in the systematic review, of which 6 were included in the meta-analysis and 13 in the descriptive synthesis. The aggregated evidence does not support clinically meaningful improvements in hard endpoints—including hospitalization and mortality—nor does it demonstrate a clear benefit in reducing PIMs. For medication adherence, the evidence remains inconclusive and highly heterogeneous, and no reliable pooled estimate could be obtained.

Taken together, the current evidence base remains insufficient to justify broad implementation of digital or AI/ML-based medication management systems in routine geriatric care, given the considerable heterogeneity, modest sample sizes across studies, and marked variability in intervention protocols. Future investigations should prioritize adequately powered, longer-term RCTs with more homogeneous populations and standardized intervention frameworks. A particular emphasis should be placed on closing the translational gap between detecting medication-related problems and achieving tangible clinical benefits, which is a critical step if digital or AI/ML-based tools are to move beyond experimental settings into real-world practice.

## Data Availability

The original contributions presented in the study are included in the article/supplementary material, further inquiries can be directed to the corresponding author.
